# Supplementary material for: Vitamin D Compounds PRI-2191 and PRI-2205 Enhance Anastrozole Activity in Human Breast Cancer Models
Source: Int J Mol Sci. 2021 Mar 9;22(5):2781. doi: 10.3390/ijms22052781 (PMC7967212; doi:10.3390/ijms22052781)
Supplement: Supplementary file 1 [file ijms-22-02781-s001.pdf]

Vitamin D Analogs Enhance Anastrozole Activity in Human Breast Cancer Models

Beata Filip-Psurska [1,*], Mateusz Psurski[1], Artur Anisiewicz[1], Patrycja Libako[1], Ewa Zbrojewicz[1], Magdalena Maciejewska[1], Andrzej Kutner[2] and Joanna Wietrzyk[1].

1 Department of Experimental Oncology, Hirszfeld Institute of Immunology and Experimental Therapy, Polish Academy of Sciences, Wroclaw, Poland
2 Pharmaceutical Research Institute, Faculty of Pharmacy, The Medical University of Warsaw, Warsaw, Poland
* beata.filip-psurska@hirszfeld.pl

1. Supplementary Results

Supplementary Table S1. Breast cancer cell lines proliferation inhibition after treatment with vitamin D analogs

	**MCF-7**	**T47D**	**SKBR-3**	**MDA-MB-231**	**MCF10A***
**Compound**	% proliferation inhibition (mean ± SD)				
**Calcitriol**	47,7±2,9	53,1±0,3	20,2±5,2	4,2±0,35	6,0 ± 3,3
**PRI-2191**	49,4±5,2	58,6±1,2	24,3±4,1	6,1 ± 2,1	2,1±3,0
**PRI-2205**	21,5±9,5	26,9±5,4	7,4±2,9	6,5 ± 2,3	0±0
**Anastrozole**	43,89 ± 5,69	37,24 ± 5,11	25,51 ± 3,21	14,26±5,03	4,2±3,9
**Anastrozole**					
**+ Calcitriol**	73,64 ± 7,76[a]	54,23 ± 3,68	46,2 ± 10,58	27,92±1,07	16,2±0,7
**+ PRI-2191**	76,29 ± 6,18[a]	59,04 ± 4,20	50,45 ± 3,01	28,42±3,63	19,6±1,4
**+ PRI-2205**	62,10 ±14,03[a]	47,94 ± 3,16[b]	43,93 ± 4,43	22,52±3,98	11,6±5,7

* MCF-10A cell line is a is a non-tumorigenic epithelial cell line, here used as a control. Calcitriol, PRI-2191 and PRI-2205 were used at the concentration of 100nM with the exception of T47D cell line, where the concentration 10nM was used. Anastrozole was applied at the dose of 0.1mg/ml. Data represent mean values ± SD of at least three independent experiments. Statistical analysis was calculated using non-parametric Kruskal-Wallis ANOVA followed by Dunn's multiple comparisons test (*$p < 0.05$, **a**- when compared to anastrozole alone, **b** – when compared to PRI-2205).

Supplementary Table S2. The expression of genes related to the estrogenic signaling pathway in MCF-7 breast cancer cells after 72h of treatment with vitamin D analogs alone or combined with anastrozole. Data presented as RQ fold change only selected genes are shown .

	Ethanol	Calcitriol	PRI-2191	PRI-2205	Anastrozole (An)	An+ calcitriol	An+PRI-2191	An+PRI-2205
**Gene**	RQ	Fold change						
**BCAR3**	1	-10,3	-7,2	+2,99	-0,5	-9,0	-2,9	-23,8
**CARM1**	1	**-10000**	**-10000**	**-10000**	**-2500**	**-10000**	**-2500,0**	**-1111,1**
**CEBPBCCAAT**	1	**-5000,0**	**-1428,6**	**-2000,0**	**-1666,7**	**-476,2**	**-1000,0**	**-1666,7**
**CYP19A1**	1	-6,7	-4,6	-4,9	-1,6	-5,8	-1,9	-15,5
**ESR1**	1	**-555,6**	**-105,3**	**-69,0**	**-59,9**	**-476,2**	**-54,3**	**-526,3**
**ESR2**	1	-11,1	-3,5	-8,2	-2,6	-9,7	-3,1	-23,8
**ESRRA**	1	-6,7	-4,6	-4,9	-1,6	-5,8	-1,9	-15,5
**ESRRG**	1	+1,38	-4,6	-4,9	-1,6	-5,8	-1,9	-15,5
**FSHB**	1	-6,7	-2,2	-4,9	-1,6	-5,8	-1,9	-15,5
**FSHR**	1	-6,7	**+340699,5**	-4,9	-1,6	-5,8	-1,9	-15,5
**GABARAPL1**	1	-12,6	-8,8	-9,3	-3,0	-11,0	-3,5	-29,2
**GNRH1**	1	-8,5	-5,9	+1,09	-2,1	-7,5	-2,4	-19,8
**GNRH2**	1	**+9,1**	-0,1	**+10,6**	**+1592,1**	-6,4	**+37,3**	+1,87
**HMGA1**	1	-13,6	-20,7	-10,2	-2,8	-26,0	-8,4	-49,3
**HSD17B2**	1	-6,7	-4,6	+3,24	-1,6	-1,7	-1,9	-15,5
**HSD17B8**	1	-6,7	-4,6	-4,9	-1,6	-5,8	-1,9	-15,5
**HSPB8**	1	-6,4	-6,9	-7,3	-2,4	-3,4	-2,8	-23,0
**IL1A**	1	-21,3	-2,1	-3,8	-5,2	-18,7	-6,0	-17,2
**INHA**	1	**-1000,0**	**-58,5**	**-200,0**	**-555,6**	**-212,8**	**-114,9**	**-625,0**
**ISG20**	1	-7,7	-3,1	**+13,5**	+7,6	-6,7	-0,4	-14,3
**LHCGR**	1	-6,7	-4,6	-4,9	-1,6	-5,8	-1,9	-15,5
**MKNK2MAP**	1	-8,3	-17,2	-8,1	-833,3	-33,0	-5,6	-1250,0
**MPGN**	1	**-1000,0**	**-666,7**	**-714,3**	**-232,6**	**-833,3**	**-270,3**	**-2500,0**
**NCOA1**	1	**-106,4**	**-74,1**	**-78,7**	-25,8	**-93,5**	-4,8	**-250,0**
**NCOA3**	1	-33,7	**-87,7**	**-151,5**	**-1428,6**	-23,0	-2,0	**-5000,0**
**NFKB1**	1	-6,7	-4,6	-4,9	-1,6	-5,8	-1,9	-15,5
**NR0B1**	1	+4,74	-4,6	-4,9	-1,6	-5,8	+1,75	-15,5
**NR2C2**	1	**-666,7**	**-1000,0**	**-10000,0**	**-5000,0**	**-10000**	**-5000,0**	0,0
**PGR**	1	**-208,3**	**-144,9**	**-153,8**	**-50,3**	**-181,8**	**-58,5**	**-476,2**
**POU4F1POU**	1	+5,94	-4,6	-4,9	-1,6	-5,8	+1,76	-15,5
**RERG**	1	-23,5	**-135,1**	**-60,2**	**-105,3**	-7,2	**-94,3**	-48,3
**SREBF1**	1	-5,2	-5,4	-5,7	-1,9	-6,7	-2,2	-17,9
**SULT1E1**	1	-9,9	-4,4	-7,3	-2,4	-8,7	-2,8	-22,9
**TAF10**	1	-16,1	-36,0	**-55,9**	**-87,7**	-15,2	**-72,5**	**-101,0**
**TCF7**	1	-11,3	-16,4	-11,4	-6,3	-22,0	-9,5	-50,5
**TFF1**	1	-8,0	-4,7	-5,8	-4,5	-8,7	-4,0	-6,1
**TRIM25**	1	-20,7	**-68,5**	-12,3	-30,0	-49,3	**-86,2**	**-116,3**

The Biomol GmbH Human Estrogen Signaling Primer Library was used, Cat. No: HESR-1- a set of commercial primers used to analyze the expression of the examined genes in MCF-7 breast cancer cells.

Real Time PCR method and comparative analysis using the ΔΔ Ct method was performed. The table shows a fold expression of given genes in relation to the calculated RQ value. The Hprt1 gene was selected as the control gene because it was characterized by a relatively low variation in expression between the tested samples and a low "score" coefficient determined in the Data Assist analysis software. All groups were compared to the ethanol control, i.e. cells grown in the medium with the addition of ethyl alcohol at a concentration corresponding to the highest concentration of this solvent used in the test, i.e. approx. 1.1% ethanol in the culture medium. Values marked with "+" signify an increase and "-" a decrease in transcription. Values in bold red type indicate a significant increase in gene expression relative to the control. Values that indicate a significant reduction in gene expression in relation to the control are marked in bold black font.
